# Brucellosis Presenting as Cholecystitis: A Case Report and Literature Review

**DOI:** 10.1093/ofid/ofz334

**Published:** 2019-07-15

**Authors:** Lisa Speiser, Lanny Hsieh, Susan S Huang, Cassiana Bittencourt, Donald Forthal

**Affiliations:** University of California Irvine

**Keywords:** brucella, brucellosis, cholecystitis, human, intra-abdominal

## Abstract

Brucellosis is a zoonotic disease endemic to much of the world. It most often presents with nonspecific symptoms and is a well known cause of undulant fever. Focal forms occur less frequently, with osteoarticular complications being the most common. In this study, we describe a rare case of brucellosis presenting as cholecystitis.

Brucellosis, caused by an aerobic, intracellular Gram-negative coccobacilli of the genus *Brucella*, often presents with nonspecific findings of intermittent fever. Focal forms are found in 30% of patients with osteoarticular manifestations as the most common [1, 2]. We present a case of brucellosis cholecystitis and review the literature on this rare presentation.

## CASE REPORT

A 57-year-old Egyptian male chef and avid eater of unpasteurized cheese presented with right upper quadrant abdominal pain in March 2018. White blood cell count (WBC) and liver function tests were normal. Ultrasound in the emergency department (ED) revealed a stone in a contracted gallbladder. Cholecystectomy was deferred due to uncertainty if the ultrasound findings were the true cause of his pain. Because of continued pain, he made repeated visits to the ED until November 2018 when he underwent laparoscopic cholecystectomy. Surgery was complicated by severing of an accessary hepatic artery and a bile leak. After an unsuccessful endoscopic retrograde cholangiopancreatography (ERCP) on postoperative day (POD) 2, he was transferred to an academic medical center due to concerns for common bile duct injury.

Upon transfer, ERCP demonstrated a truncated common bile duct, and an angiogram confirmed ligation of the right hepatic artery. White blood cell count was 9600, alkaline phosphatase (ALP) 69, alanine aminotransferase (ALT) 160, aspartate aminotransferase (AST) 79, and bilirubin (TBILI) 4.5. Blood cultures (BCs) obtained on POD 3 were negative. After stabilization and supportive care, he was discharged on POD 5 with plans for drain placement by interventional radiology and definitive surgery by hepatobiliary in 6 weeks to achieve sufficient collateral formation.

On POD 11, the patient developed fevers, abdominal pain, and jaundice. He was found to have a WBC of 27 000, ALP 255, ALT 432, AST 320, and TBILI 10.7. A computerized tomography scan of the abdomen showed a large biloma above the right lobe of the liver extending laterally, medially, and inferiorly. The biloma was aspirated percutaneously and drains were placed. Cultures from the aspiration and from 1 set of BC obtained at admission grew *Brucella melitensis*. A serum *Brucella* titer, obtained POD 18, was 1:80. He was initiated on doxycycline, and, because streptomycin is not available in the United States, he was given a short 1-week course of gentamicin [3] to avoid renal toxicity while awaiting surgical correction.

On POD 35, he underwent biliary reconstruction with Roux-en-Y hepaticojejunostomy. Intraoperatively, he was noted to have abscess cavities as well as necrotic and purulent material in the gallbladder fossa. Cultures were negative. Postoperatively, he continued doxycycline for 6 weeks and was reinitiated on gentamicin to complete a 2-week course of gentamicin from the time of corrective surgery. His abdominal pain and dyspepsia completely resolved and he remains asymptomatic 4-months later.

Lack of suspicion for brucellosis led to laboratory exposure work-up of 25 employees. Six of seven high-risk exposures received antimicrobial postexposure prophylaxis (1 declined). Per guidelines [4], all exposed workers were monitored serologically and for symptoms. None seroconverted or developed brucellosis.

## DISCUSSION AND LITERATURE REVIEW

To date, there have been 26 cases of *Brucella* cholecystitis reported in the literature (Supplementary Table 1). Our patient’s clinical syndrome was assumed to be due to consumption of unpasteurized cheese, where ingested *Brucella* invades the mucosa and is taken up by local macrophages [5–8]. Here, it remains intracellularly and forms membrane-bound compartments [5, 7–10]. The intravesicular pH acidifies and allows *Brucella* to evade antimicrobials that lack activity in an acidic environment [5–8, 10]. The *Brucella* bacteria replicates and establishes a latent infection that escapes the immune system for years [7, 9, 11]. We postulate that gallstone-induced cholecystitis allowed inflammation to reactivate latent brucellosis, which was amplified by inflammation and pooling of nutritive blood and bile due to the transection of the bile duct and hepatic artery in his original surgery. This is consistent with *Brucella*’s incubation period of 1 to 4 weeks [12].

Treatment of brucellosis consists of a combination of antibiotics with activity in acidic intracellular environments such as the following: tetracyclines, aminoglycosides, and rifampin [13]. Secondary therapies include fluoroquinolones and trimethoprim/sulfamethoxazole [7]. Monotherapy is associated with a relapse rate of 10%–20% [5, 13]. The treatment for complicated, localized disease has not been well established, and the optimal duration of treatment is unknown [1]. In the case reports reviewed here, duration of therapy ranged from 8 days to 6 months, with the most common treatment regimen of streptomycin or gentamicin plus doxycycline for 6 weeks. Three cases mention asymptomatic follow-up at 6 weeks [14], 2 months [1], and 90 days [15] after 6 weeks, 3.5 months, and 2.5 months of treatment, respectively.

## CONCLUSION

We report an unusual case of brucellosis presenting as cholecystitis after instrumentation and injury in a patient from an endemic region. Lack of suspicion of this rare entity necessarily resulted in a laboratory exposure work-up.

## Supplementary Data

Supplementary materials are available at *Open Forum Infectious Diseases* online. Consisting of data provided by the authors to benefit the reader, the posted materials are not copyedited and are the sole responsibility of the authors, so questions or comments should be addressed to the corresponding author.

ofz334_suppl_supplementary_material

## References

[1] Starakis I, Polyzogopoulou E, Siagris D, et al Unusual manifestation of brucellosis: liver abscess and pancytopenia caused by *Brucella melitensis*. Eur J Gastroenterol Hepatol 2008; 20:349–52. 18334880 10.1097/MEG.0b013e3282ef9493

[2] Miranda RT, Gimeno AE, Rodriguez TF, et al Acute cholecystitis caused by *Brucella melitensis*: case report and review. J Infect 2001; 42:77–8. 11243761 10.1053/jinf.2000.0757

[3] Hasanjani Roushan MR, Mohraz M, Hajiahmadi M, et al Efficacy of gentamicin plus doxycycline versus streptomycin plus doxycycline in the treatment of brucellosis in humans. Clin Infect Dis 2006; 42:1075–80. 16575723 10.1086/501359

[4] Centers for Disease Control and Prevention. Assessing laboratory risk level and PEP. Available at: https://www.cdc.gov/brucellosis/laboratories/risk-level.html. Accessed 9 July 2019.

[5] Arenas GN, Staskevich AS, Aballay A, Mayorga LS Intracellular trafficking of *Brucella abortus* in J774 macrophages. Infect Immun 2000; 68:4255–63. 10858243 10.1128/iai.68.7.4255-4263.2000 PMC101738

[6] Pappas G, Akritidis N, Bosilkovski M, Tsianos E Brucellosis. N Engl J Med 2005; 352:2325–36. 15930423 10.1056/NEJMra050570

[7] Franco MP, Mulder M, Gilman RH, Smits HL Human brucellosis. Lancet Infect Dis 2007; 7:775–86. 18045560 10.1016/S1473-3099(07)70286-4

[8] Liautard JP, Gross A, Dornand J, Köhler S Interactions between professional phagocytes and *Brucella* spp. Microbiologia 1996; 12:197–206. 8767704

[9] Delrue RM, Lestrate P, Tibor A, et al *Brucella* pathogenesis, genes identified from random large-scale screens. FEMS Microbiol Lett 2004; 231:1–12. 14979322 10.1016/S0378-1097(03)00963-7

[10] Lapaque N, Moriyon I, Moreno E, Gorvel JP *Brucella* lipopolysaccharide acts as a virulence factor. Curr Opin Microbiol 2005; 8:60–6. 15694858 10.1016/j.mib.2004.12.003

[11] Forestier C, Moreno E, Pizarro-Cerda J, Gorvel JP Lysosomal accumulation and recycling of lipopolysaccharide to the cell surface of murine macrophages, an in vitro and in vivo study. J Immunol 1999; 162:6784–91. 10352299

[12] Mantur BG, Amarnath SK, Shinde RS Review of clinical and laboratory features of human brucellosis. Indian J Med Microbiol 2007; 25:188–202. 17901634 10.4103/0255-0857.34758

[13] WHO/CDS/EPR/2006.7. Brucellosis in Humans and Animals. Geneva: World Health Organization, 2006.

[14] Al-Otaibi FE Acute acalculus cholecystitis and hepatitis caused by *Brucella melitensis*. J Infect Dev Ctries 2010; 4:464–7. 20818096 10.3855/jidc.618

[15] Gunal E, Topkaya A, Arisoy A, et al Cholecystitis related to *Brucella melitensis* a rare presentation. Infect Dis Clin Pract 2008; 16:134–6.

