# Supplementary material for: Brucellosis Presenting as Cholecystitis: A Case Report and Literature Review
Source: Open Forum Infect Dis. 2019 Jul 15;6(10):ofz334. doi: 10.1093/ofid/ofz334 (PMC6765347; doi:10.1093/ofid/ofz334)
Supplement: ofz334_suppl_supplementary_material [file ofz334_suppl_supplementary_material.docx]

**SUPPLEMENTAL SECTION: TABLE 1**

| **Source** | **Date** | **Patient** | **Gallbladder culture** | **Blood culture** | **Surgical removal** | **Brucella Titers** | **Antibiotic tx and duration** |
| --- | --- | --- | --- | --- | --- | --- | --- |
| Bull P et al. Norsk Magazin for Laegevidenskaben 1911; 72: 1026 | 1911 | 43 F | B. melitensis | NA | Yes | NA | None |
| Leavel HR. et al. Am J Med Sci 1931; 181: 96 | 1931 | 53 M | Brucella spp. | Negative | Yes | NA | None |
| Amoss HL. Internat Clin 1931; 4: 93–8 | 1931 | 48 F | Brucella spp. | Brucella Spp | Yes | NA | None |
| Amoss HL. Internat Clin 1931; 4: 93–8 | 1931 | 45 M | Brucella spp. | NA | Yes | NA | None |
| White CS. Med Ann DC 1934; 12: 60–2 | 1934 | ? F | Brucella spp. | NA | NA | NA | None |
| Mettier SR. Arch Intern Med 1934; 54: 702–9 | 1934 | 57 M | B. melitensis | B. melitensis | Yes | NA | None |
| Hewlett JS et al. Cleve Clin Q 1947; 24: 258–63 | 1947 | 58 M | B. abortus | Negative | No | NA | Streptomycin x 8 days |
| Valenzuela CE. Rev Esp Enf Ap Digest 1971; 33: 723–6 | 1971 | 56 M | NA | NA | Yes | 1:400 | Tetracycline + streptomycin x NA |
| Morris SJ et. al. Am J Gastroenterol 1979; 71: 481–4 | 1979 | 34M | B. suis | B. suis | Yes | 1:1280 | Tetracycline + streptomycin x 3 weeks |
| Berbegal JM et al. Rev Esp Enf Ap Digest 1986; 70: 88 | 1986 | 33 M | Negative | B. melitensis | Yes | 1:320 | Tetracycline + streptomycin x NA |
| Shaheen SE. et al. Am J Gastroenterol 1989; 84: 336–7 | 1989 | 42 F | B. melitensis | B. melitensis | Yes | 1:640 | Tetracycline + streptomycin x 3 weeks |
| Colmenero J. et al. Medicine (Baltimore) 1996;75:195-211. | 1996 | 58 M | Negative | B. melitensis | Yes | 1:160 | Doxycycline + streptomycin x 3 weeks |
| Fasquelle D, et. al. Eur J Clin Microbiol Infect Dis 1999; 18: 599–600 | 1999 | 72 F | B. melitensis | B. melitensis | Yes | 1:80 | Doxycycline + rifampin x 6 weeks |
| Serrano FJ, et. al. Rev Clin Esp 1999; 199: 621–62 | 1999 | 59 M | Negative | B. melitensis | Yes | 1:1000 | Doxycycline + streptomycin x NA |
| Ashley D, et. al. Pediatr Infect Dis J 2000; 19: 1112–3 | 2000 | 6 M | NA | B. abortus | No | NA | TMP/SMX + rifampin x 6 weeks |
| Miranda R, et. al. J Inf 2001; 42: 77-78. | 2001 | 34 M | B. melitensis | Negative | Yes | 1:2560 | Doxycycline + rifampin x 6 weeks |
| Andriopoulos P., et. al. Scand J Infect Dis 2003; 35: 204–5 | 2003 | 72 M | B. melitensis | B. melitensis | Yes | NA | Doxycycline + streptomycin x 6 months |
| Lopez-Prieto MD, et. al. Enferm Infec Microbiol Clin 2003; 21: 464–5 | 2003 | 56 F | B. melitensis | Negative | Yes | NA | Doxycycline + streptomycin x 6 weeks |
| Kanafani Z, et. al. Scandinavian Journal of Infectious Diseases 2005; 37: 927-930. | 2005 | 55 M | Brucella spp. | Brucella spp. | Yes | 1:80 and 1:160 | Doxycycline + rifampin x 6 weeks |
| Kanafani Z, et. al. Scandinavian Journal of Infectious Diseases 2005; 37: 927-930. | 2005 | 29 F | Brucella spp. | Negative | Yes | 1:80 and 1:640 | Doxycycline + rifampin x 6 weeks |
| Pourbagher MA, et. al. AJR 2006; 187: W191-W194. | 2005 | NA | No culture | NA | Yes | Elevated, not defined | Ciprofloxacin + tetracycline + rifampicin x 45 days |
| Al-Anazi K, et. al. J Med Case Rep 2007; 1:144. | 2007 | 54 M | NA | B. melitensis | No | 1:2560 | Netilmicin + Doxycycline x 3 weeks followed by Ciprofloxacin + Doxycycline x 3 weeks |
| Starakis I, et. al. Eur J of Gastro & Hep 2008; 20 (4): 349-352. | 2008 | 54 M | Negative | B. melitensis | yes | Negative | Doxycycline + rifampicin + gentamicin x 14 days followed by Docycycline + rifampicin x 3 months |
| Gunal E, et al. Infect Dis Clin Pract 2008; 16: 134-136. | 2008 | 64 F | NA | B. melitensis | No | 1:80 | Streptomycin + Doxycycline + Rifampicin x 14 days followed by Doxycycline + Rifampicin x 2 months |
| Al Otaibi F, et. al. J Infect Dev Ctries 2010; 4: 4464-467. | 2010 | 42 M | NA | B. Melitensis | No | 1:80 and 1:2560 | Doxycycline x 6 weeks + streptomycin x 2 weeks |
| Ali B. Ann Clin Case Rep 2016; 1: 1101. | 2016 | 40 M | NA | Brucella Spp. | No | 1:2560 | Streptomycin + Doxycycline x 6 weeks |
| Speiser L, et al. | 2019 | 57 yo | B. melitensis | B. Melitensis | yes | 1:80 | Doxycycline – 6 weeks + Gent – 2 weeks |
